# From education to exploitation: the high price paid by resident physicians in Ecuador's medical specialization

**DOI:** 10.3389/fmed.2024.1409471

**Published:** 2024-11-21

**Authors:** Juan S. Izquierdo-Condoy, Carlos Ruiz-Sosa, Andrea Tello-De-la-Torre, Esteban Ortiz-Prado

**Affiliations:** One Health Research Group, University of the Americas, Quito, Ecuador

**Keywords:** medical specialization, resident physicians, workforce, systemic exploitation, health policy

## Introduction

The evolving landscape of healthcare necessitates a critical examination of medical training and specialization, particularly within the context of Ecuador. As medicine advances rapidly across various disciplines, the selection of a medical specialty has transformed from an option to a necessity for medical graduates. Aspiring specialists must now undergo a postgraduate program or medical residency, defined as entry into a medical unit for the purpose of specialization. This period is characterized by the development of complex professional activities that require increasing responsibility. These activities must be completed within the time frame specified by the corresponding academic and operational programs, with courses endorsed and supervised by a university institution (1).

**Figure 1:**
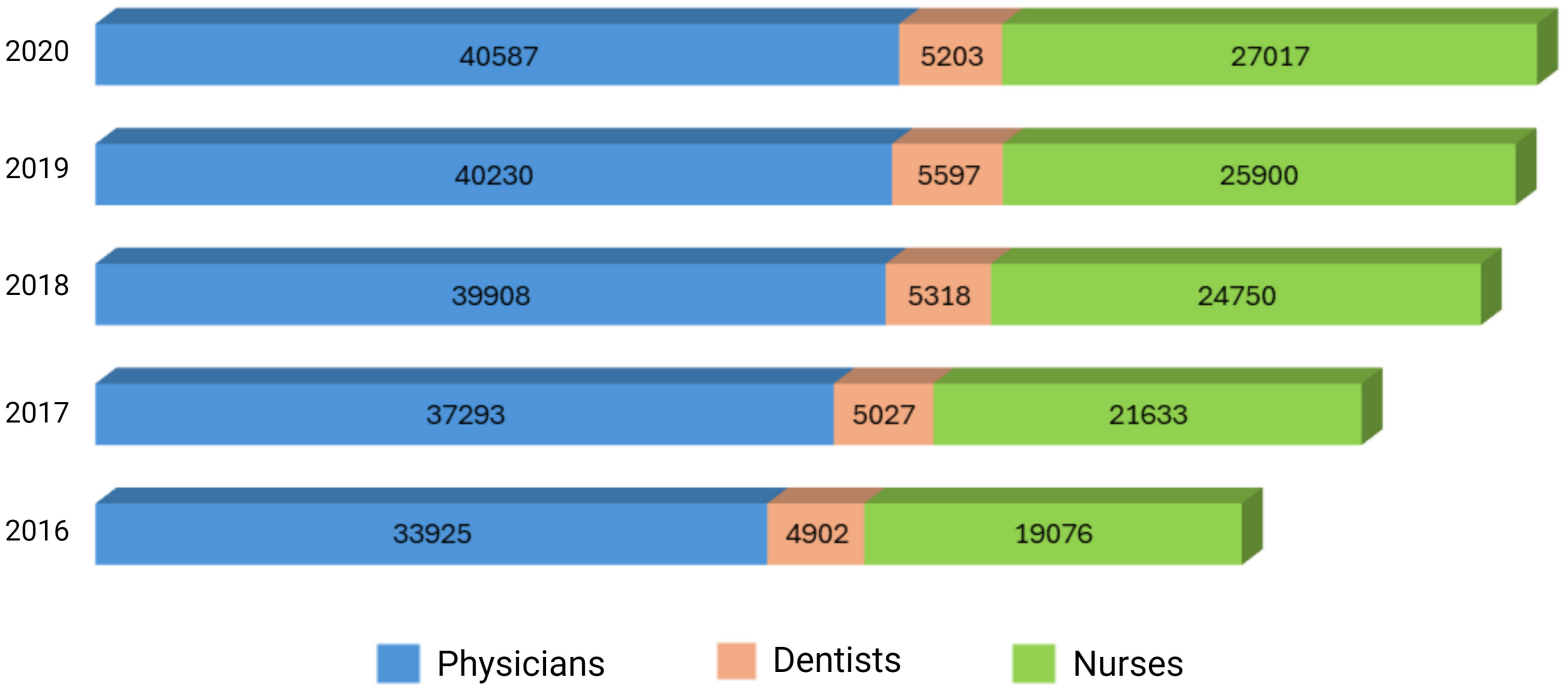
Proportion of health professionals (physicians, dentists, and nurses) in Ecuador from 2016 to 2020.

In Ecuador, university training in the field of health has developed within a framework of growth, particularly over the last few decades, as more universities are able to train health professionals. Despite the predominance of physicians over other professions such as dentists and nurses, official data from the National Institute of Statistics and Censuses (INEC) show a decrease in the proportion of physicians, from 58.6% (*n* = 33,925) in 2016 to 55.7% (*n* = 40,587) in 2020. This decline is mainly attributed to an increase in the proportion of nursing professionals, which rose from 32.9% (*n* = 19,076) in 2016 to 37.1% (*n* = 27,017) in 2020 (2) (Figure 1). This trend indicates a clear diversification of the health workforce, reflecting the perspectives of aspiring health professionals regarding educational programs and career opportunities in Ecuador.

**Figure 2:**
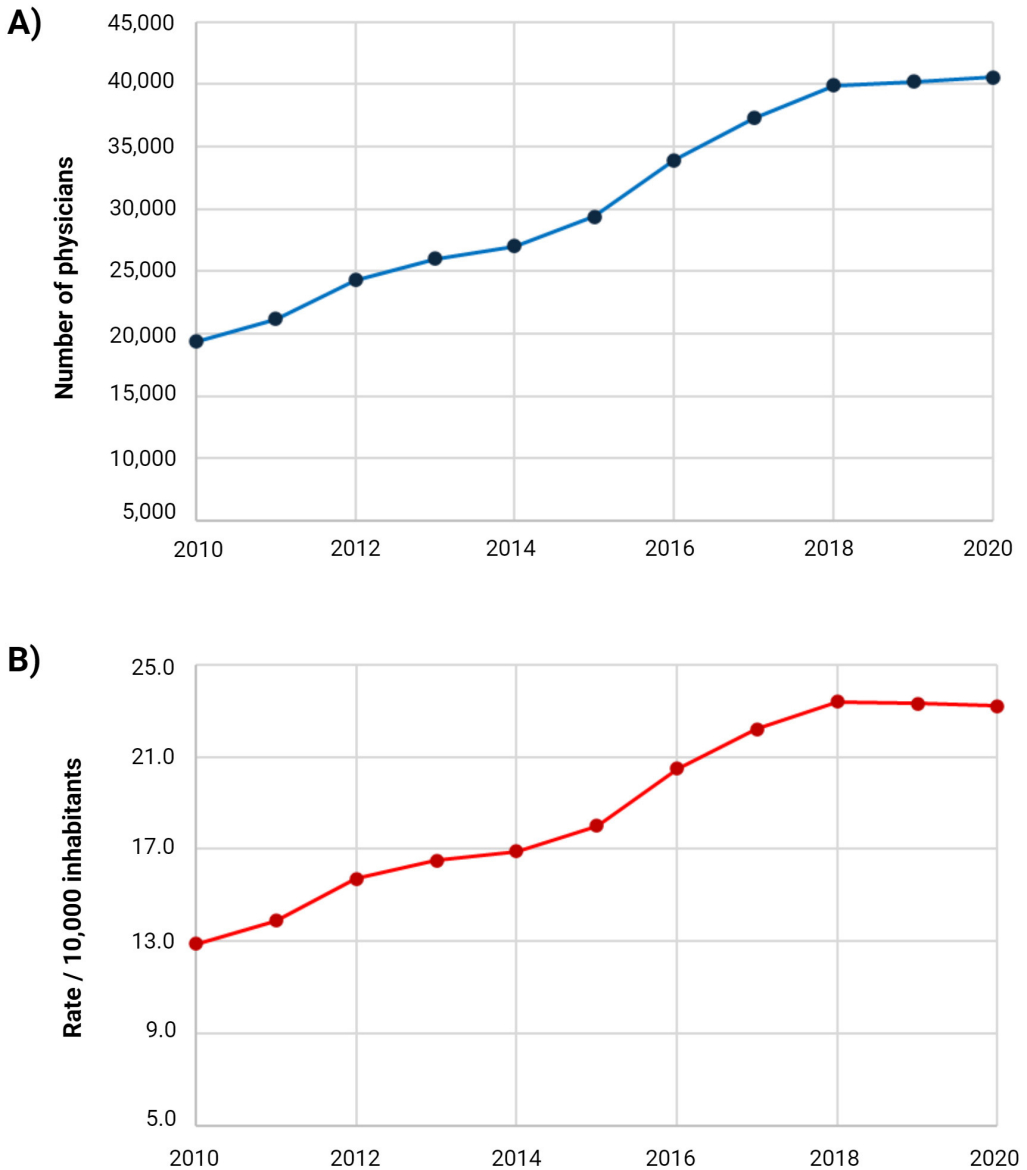
Temporal evolution of physicians in Ecuador from 2010 to 2020. **(A)** Number of physicians for each year between 2010 and 2020; **(B)** rate of physicians per 10,000 inhabitants from 2010 to 2020.

Furthermore, an analysis focused on physicians reveals that over the last decade, the number of physicians has significantly increased, with the total in 2020 (*n* = 40,587) doubling that in 2010 (*n* = 19,344). The rates increased from 12.9 physicians per 10,000 inhabitants in 2010 to 23.2 per 10,000 inhabitants in 2020 (2) (Figure 2). Although Ecuador reached the World Health Organization's minimum recommendation of 23 physicians per 10,000 inhabitants in 2018, there remains a significant shortage of specialist physicians (3, 4). This deficiency has important implications for the overall effectiveness of the health system. Consequently, authorities and several universities have proposed measures to promote medical specialty programs and increase the number of specialist physicians (5).

It is essential to emphasize that although resident doctors are in a training phase, they hold a university degree. Apart from academic commitments, they also perform healthcare-related tasks, encompassing both medical and surgical duties. These resident physicians significantly contribute to the global healthcare workload and command a substantial share of budget allocations from regulatory bodies (6).

Given the vital role that this population plays in healthcare systems, it is imperative for responsible institutions to establish residency programs that not only advance professional competence but also ensure the wellbeing and adequate living conditions of the residents. While the cultural elements of medical residency programs may vary by country, common factors such as access, compensation, and workload provide a basis for quality assessment (6–8).

In Ecuador, aspiring medical professionals typically gain access to specialized programs either through scholarships or by self-financing their education. However, both pathways present significant challenges. While scholarships are valuable, they often fail to cover the full costs associated with medical training, leading to financial shortfalls (9). Conversely, the self-financing option is prohibitively expensive, with average costs ranging from $20,000 to 40,000. This financial burden is particularly daunting for medical graduates who find themselves unemployed upon completing their degrees. The economic strain is exacerbated for those who are already supporting families, casting a shadow over their financial stability and wellbeing (10).

As delineated by the Higher Education Council of Ecuador's Technical Standard for Specialization in the Field of Health, resident doctors are categorized as “students in training.” Consequently, they fall outside the public health sector's salary structures and are ineligible for regular employee remuneration. While they may be entitled to certain social security benefits, they do not receive salaries or any other forms of financial compensation. This classification subjects them to a form of systemic exploitation, where they are frequently overworked and underappreciated, embodying a workforce that endures ongoing abuse and excessive utilization (11). Building on the previous discussion about the challenges faced by resident doctors in Ecuador, it's important to highlight that despite not being recognized as workers within the public health sector, these residents are subject to rigorous and well-defined work schedules. The Higher Education Council of Ecuador mandates that their weekly schedule includes 64 h dedicated to patient care and an additional 16 h for academic classes. These medical residents are required to fulfill a specified minimum of daily care hours, along with completing 24-h shifts approximately every 4 days. Notably, this demanding schedule includes weekends and holidays, with no exemptions. Consequently, it's not uncommon for residents to work upwards of 100 h per week, significantly surpassing the already stringent guidelines. This intense workload, coupled with their status as “students in training”—which precludes them from salary structures and limits their compensation primarily to some social security benefits—highlights the systemic exploitation and excessive demands placed on these individuals, exacerbating the challenges they face in their pursuit of medical specialization (12, 13). Personal testimonies from residents highlight the difficulties of sustaining oneself without income over 3–4 years, especially for those with families and limited resources to support their academic training (9, 14).

The geographic distribution of available positions in medical specialization programs in Ecuador requires many residents to train in provinces distant from major urban centers, such as Quito and Guayaquil. This distribution forces residents to relocate to areas with limited infrastructure and fewer technological resources, which are crucial for their training. Consequently, most medical residents aspire to return to larger cities after completing their studies to work in hospitals with greater complexity, where they can pursue subspecialization and professional growth. This concentration of opportunity restricts their professional trajectories and often delays their goals for advanced training in environments offering broader academic and clinical resources (15).

Another significant challenge for resident doctors is the scarcity of specialized medical programs outside major cities, leading to centralized healthcare resources and resulting in disparities in specialist availability. While some cities meet or exceed global standards for specialists per capita, others lack specialists entirely (16). This issue of geographic inequality in healthcare access and specialist distribution is not unique to Ecuador. In Pakistan, for example, a similar centralized distribution model places specialized hospitals in major urban centers, such as Islamabad, Lahore, and Karachi, leaving rural areas underserved. Additionally, resident doctors in Pakistan often face the challenge of working without salary compensation during their training, despite familial responsibilities (17).

Research underscores the significant health risks associated with the demanding work schedules of resident doctors, noting that workweeks exceeding 80 h can have serious detrimental effects on their wellbeing, including depression, anxiety, and burnout syndrome (18–20). The prevalence of elevated stress levels among resident doctors is a global phenomenon, which raises concerns about the impact of such stress on the quality of patient care and satisfaction (6). These findings should serve as a call to action for responsible institutions to address the issue, as the wellbeing of physicians is intrinsically linked to their ability to provide high-quality care. The systemic exploitation and overuse of resident doctors not only undermine their health and professional development but also pose a risk to the overall efficacy of healthcare systems worldwide (21).

To enhance the depth and impact of this analysis, incorporating perspectives from other stakeholders, such as healthcare administrators, policymakers, and patients, could offer valuable insights. Healthcare administrators, for example, could clarify the logistical and financial constraints of implementing changes, while policymakers could address regulatory challenges. Patient perspectives could further clarify how residents' working conditions influence the quality of care, enriching the debate on healthcare outcomes. A multi-stakeholder approach could foster collaboration across different sectors to develop solutions benefiting both resident physicians and the healthcare system. Future research should consider these additional viewpoints to ensure reforms align with the needs of all healthcare stakeholders.

Although the current status of resident physicians in Ecuador remains unclear, research from the past decade has consistently revealed adverse conditions, including overwork, inadequate academic training, and professional burnout (22, 23). Additionally, a recent study of physicians in Ecuador's mandatory rural social service program reported low levels of overall job satisfaction (24). These findings highlight a concerning trend within the Ecuadorian health system, suggesting neglect and abuse of medical personnel in training stages.

In this context, it is urgent to improve the conditions for resident physicians in Ecuador. Priority initiatives should include adequate remuneration for basic subsistence, regulated working hours to protect resident welfare, and provisions for both academic and professional development (25). Successful models from other countries in the Americas and beyond could provide valuable guidance. For example, the United States, through the Accreditation Council for Graduate Medical Education (ACGME), has implemented wellness-promoting standards that include work-hour restrictions and access to mental health resources (26). Canada's residency programs incorporate the CanMEDS Framework, which promotes balanced professional development and personal wellness (27).

In Europe, Germany's residency programs provide competitive salaries, enforce reasonable work hours, and include comprehensive health insurance coverage, ensuring both the financial stability and health of resident physicians (28). Australia addresses healthcare disparities by offering significant financial incentives to residents training in rural and underserved areas, thereby increasing hands-on experience while mitigating rural workforce shortages. Additionally, Australian hospitals support mental health and wellbeing through confidential counseling services and workshops on stress management and resilience building (29).

Recognizing the pivotal role of resident doctors, we strongly encourage government bodies, healthcare institutions, academic organizations, and civil society to collaborate in enacting meaningful reforms that address the pressing challenges faced by resident doctors in Ecuador and other countries with similar conditions (1). A collective effort should aim to establish comprehensive regulations for fair compensation, manageable working hours, and equitable treatment for residents, alongside institutional support that prioritizes their welfare, mental health services, and career development opportunities.

## Conclusions

Improving the conditions of resident physicians in Ecuador is essential to building a resilient healthcare system. We advocate for reforms that challenge norms devaluing their contributions, ensure transparency in postgraduate position allocation, and address systemic issues such as excessive work hours, limited financial support, and geographic disparities that hinder residents' professional growth and wellbeing. Professional associations should strengthen advocacy efforts, while civil society raises awareness of the essential role resident physicians play. Drawing on successful international models, Ecuador can implement changes that provide fair compensation, regulated work hours, and mental health support, improving both resident wellbeing and healthcare delivery. A collaborative approach involving government agencies, healthcare institutions, and academic organizations is crucial to aligning residency programs with the broader needs of the healthcare system and its stakeholders. By supporting and valuing resident physicians, we invest in the future of healthcare, promoting a system that treats them with the dignity and respect they deserve in their critical role.
